# Association between Alanine Aminotransferase and Growth Hormone: A Retrospective Cohort Study of Short Children and Adolescents

**DOI:** 10.1155/2019/5939372

**Published:** 2019-04-03

**Authors:** Baolan Ji, Mei Zhang, Qianqian Zhao, Yuntian Chu, Yanying Li, Hui Pan, Bo Ban

**Affiliations:** ^1^Department of Endocrinology, Affiliated Hospital of Jining Medical University, Jining Medical University, Jining, Shandong 272029, China; ^2^Chinese Research Center for Behavior Medicine in Growth and Development, Jining, Shandong 272029, China; ^3^School of Health Management and Medicine, Tongji Medical College, Huazhong University of Science and Technology, Wuhan, Hubei 430030, China; ^4^Key Laboratory of Endocrinology of National Health and Family Planning Commission, Department of Endocrinology, Peking Union Medical College Hospital, Chinese Academy of Medical Science and Peking Union Medical College, Beijing 100730, China

## Abstract

**Objective:**

This study aimed to examine the relationship between serum alanine aminotransferase (ALT) and growth hormone (GH) in children and adolescents with short stature.

**Methods:**

In this retrospective cohort study, 670 Chinese children and adolescents with short stature were included, and 253 of them received recombinant human GH (rhGH) therapy. Anthropometric and biochemical indicators were measured. GH peak levels were assessed after provocation tests with L-dopa and insulin. The subjects were divided into 3 groups according to the GH peak level. The association between the GH peak and ALT was analyzed. The change of ALT during rhGH therapy was assessed by a generalized additive mixed model.

**Results:**

Serum ALT and incidence of ALT elevation were both decreased across the GH tertiles (*P = *0.002, 0.012, respectively). A univariate analysis showed that the GH peak was negatively associated with ALT (*β*: -0.12; 95%CI: -0.22, -0.02;* P *= 0.023). Furthermore, multiple linear stepwise regression analysis demonstrated that the GH peak was independently related to ALT after adjusting for other confounding variables (*β*: -0.12; 95%CI: -0.24, -0.00;* P* = 0.042). Besides, mean values of the change in ALT from baseline displayed that, during the early stages of rhGH treatment, serum ALT level indicated a temporary upward trend, but it subsequently gradually decreased (*β*: -0.16; 95%CI: -0.23, -0.09;* P* < 0.001).

**Conclusions:**

GH secretion level was strongly negatively correlated with ALT in short children and adolescents. And rhGH therapy could reduce ALT level over time.

## 1. Introduction

Nonalcoholic fatty liver disease (NAFLD) encompasses a large spectrum of pathological changes that includes simple mild fatty liver to nonalcoholic steatohepatitis (NASH), fibrosis, and ultimately cirrhosis in the absence of excessive alcohol consumption, which is a common, serious disease that affects the health of adults, children, and adolescents [1, 2]. Moreover, many clinical and epidemiological studies have revealed that NAFLD is associated not only with liver-related morbidity and mortality but also with an increased risk of developing cardiometabolic diseases [3].

Various diagnostic tools, including ultrasound, computed tomography (CT), and magnetic resonance (MR), can be used to evaluate NAFLD but may not be suitable for large-scale epidemiologic studies due to time consumption and cost [4, 5]. As a common and useful indicator of hepatocyte injury, elevated serum alanine aminotransferase (ALT) level is a valuable way to screen for NAFLD that reflects liver histological progression [6–9]. Moreover, research has shown that serum ALT level is independently correlated with hepatic triglyceride content and might be more appropriate for use as a predictor of the degree of NAFLD than aspartate aminotransferase and gamma-glutamyl transferase [10].

The pathogenesis of NAFLD has not yet been clearly elaborated. Growth hormone (GH) is an anterior pituitary hormone that is a key regulator of fat metabolism, and the liver is a major target of GH. Experimental research has indicated that the liver GH receptor or dysfunction of its downstream signaling pathways could elevate intracellular lipid accumulation and promote the development of NAFLD [11–13]. Additionally, clinical data have shown that GH deficiency (GHD) might be closely associated with the occurrence and progression of NAFLD [14–17].

Short stature is a condition characterized by a height more than 2 standard deviations (SD) below the corresponding mean height for a given age, sex, and population group and can significantly affect the health-related quality of life of children, adolescents, and even adults [18]. One common etiology for short stature is involved in GHD [19]. Previous studies have demonstrated that GHD is induced by various causes, such as tumors, and plays an important role in the development of NAFLD in pediatric patients [14, 15]. However, there are few studies of the relationship between GH and liver enzyme in short children and adolescents, especially when caused by GHD.

The secretory pattern of GH is pulsatile, and a single fasting GH measurement might not accurately reflect GH level. A peak-stimulated GH after stimulation testing may be more suitable to assess the body's GH secretion status. In addition, recombinant human growth hormone (rhGH) is widely used for the treatment of short stature due to GH insufficiency and other growth disorders [20]. Consequently, the present study was carried out retrospectively with the purpose of analyzing the association between serum ALT and peak-stimulated GH levels, and observing the change of ALT during rhGH therapy in Chinese children and adolescents with short stature.

## 2. Subjects and Methods

### 2.1. Subjects

This retrospective cohort study was performed by reviewing the medical records of patients with short stature from the Department of Endocrinology, Affiliated Hospital of Jining Medical University between March 2013 and July 2018. The inclusion criteria were (1) short stature, a condition characterized by a height more than 2SD below the corresponding mean height for a given age, sex, and population group; (2) normal weight and height at birth. The exclusion criteria were (1) subjects who had a history of liver disease, including viral hepatitis or genetic and autoimmune liver diseases; (2) subjects who suffered from chromosomal abnormalities, skeletal dysplasia, genetic metabolic diseases, thyroid dysfunction, and a history of use of medication that could affect GH secretion or function [21]. In all, six hundred and seventy children and adolescents with short stature and 10.2 ± 3.5 years of age were enrolled in this study. And among them, two hundred and fifty-three subjects received rhGH and were followed up.

The study was approved by the Human Ethics Committee of the Affiliated Hospital of Jining Medical University. All procedures were performed in accordance with ethical standards laid out in the Declaration of Helsinki. Written parental consent was obtained for all participants.

### 2.2. Anthropomorphic Measurements

The height of each participant was measured to the nearest 0.1 cm using the same height-measuring instrument (Nantong Best Industrial Co. Ltd., Jiangsu, China) and participants were measured without head coverings. Weight was measured using the same electronic scale (Xiangshan Weighing Apparatus Co. Ltd., Guangdong, China) in the fasting state and was accurate within ± 0.1 kg. Body mass index (BMI) was calculated with the following formula: weight (kg)/height (m^2^). Bone age (BA) was measured using an X-ray taken of the left hand, including the hand bone, wrist, and radial ulnar stem (3-4 cm). The image was scanned, and the BA was evaluated according to the Greulich-Pyle method by the same specially assigned investigator [22].

### 2.3. Laboratory Measurements

To assess GH secretion, provocation tests with L-dopa and insulin were performed. L-dopa (Levodopa Tablets®, He Feng, Guang Xi, China; dosing: body weight above 30 kg, 500 mg; below 30 kg, 250 mg) was administered orally, and insulin (Insulin Injection®, Wan Bang, Jiang Su, China, 0.1U/kg) was subcutaneously injected in the overnight fasting state. Blood samples were collected at 0, 30, 60, 90, and 120 min, respectively. GH concentration of each time point was measured using a chemiluminescence method (ACCESS2, Beckman Coulter; USA) with a sensitivity of 0.010 *μ*g/L. Serum insulin-like growth factor-1 (IGF-1) was measured by the chemiluminescence immunometric method (DPC IMMULITE 1000 analyzer, SIEMENS, Germany). Lipid profiles, including total cholesterol (TC), triglycerides (TG), high density lipoprotein-cholesterol (HDL-c), and low density lipoprotein-cholesterol (LDL-c); fasting plasma glucose (FPG) and kidney function, including Cr and uric acid (UA) were all tested using a biochemical autoanalyzer (Cobas c 702, Roche; Shanghai, China). The serum ALT level was measured with an ALT kit (IFCC enzyme colorimetric method) (Roche Diagnostics, Mannheim Germany), and the mean and variabilities of with-run precision for ALT measurements were 47.5 U/L and 2.4%. The mean and variabilities of the intermediate precision were 39.9 U/L and 1.4%.

### 2.4. Statistical Analysis

Statistical analysis was performed using R statistical software (https://www.r-project.org) and EmpowerStats (http://www.empowerstats.com, X& Y solutions, Inc. Boston MA). Continuous variables were presented as the means ± SD, and categorical variables were presented as a percentage (%). Analysis of variance test was used for comparisons of continuous variables, and chi-square test for categorical variables. Univariate and multiple linear stepwise regression analyses were used to assess the association between ALT and GH peak levels. Generalized additive mixed model was used to analyze the change in ALT from baseline during rhGH therapy over time.* P* values (2-tailed) less than 0.05 were regarded as significant differences.

## 3. Results

### 3.1. Clinical and Biochemical Characteristics

As shown in Table 1, this study enrolled 670 participants with short stature. In accordance with the literature, the normal upper limit values for serum ALT were 25.8 U/L for males and 22.1 U/L for females [6, 7]. 9.0% of the subjects had ALT elevation in the present study. In addition, the GH peak value was defined as the highest level of GH at any time point in any excitation test. The participants were divided into three groups according to the GH peak levels (Table 2). The tertiles of GH peak were defined as follows: GH < 5 ng/mL; 5 ng/mL ≤ GH < 10 ng/mL; GH ≥ 10 ng/mL. The results displayed that the BMI, TG, LDL-c, ALT and incidence of ALT elevation decreased across the tertiles (all* P* < 0.05). However, there were no obvious differences in age, SBP, DBP, TC, HDL-c, FPG, Cr and IGF-1 in the GH tertiles (all* P* > 0.05).

### 3.2. Correlation Analysis between ALT and GH Peak

Table 3 shows the correlation between the ALT and GH peak and other variables according to univariate analysis. ALT was related positively to age, BMI, SBP, DBP, TG, UA, and IGF-1 (all* P* < 0.05) and negatively to GH peak level (*P* = 0.023). However, there was no significant association between ALT and TC, HDL-c, LDL-c, FPG, and Cr (all* P* > 0.05).

Furthermore, the age, BMI, SBP, DBP, UA, TG, and IGF-1 were set as confounding variables based on the results of univariate correlation analysis. A multiple linear stepwise regression analysis was conducted in all subjects. The results showed that, after adjusting for the above confounding factors, the GH peak was independently related to the ALT (*β*: -0.12; 95%CI: -0.24, -0.00;* P* = 0.042).

### 3.3. Generalized Additive Mixed Model

**Figure 1:**
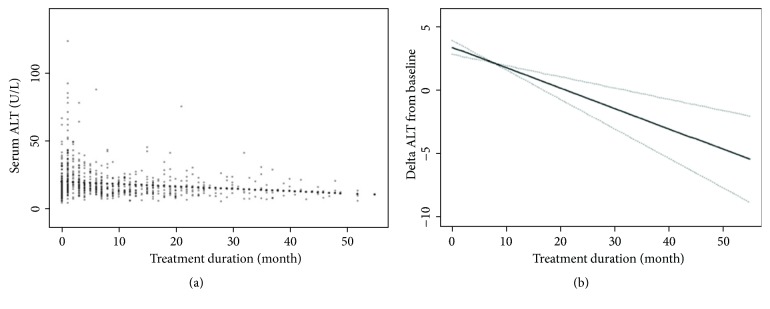
Change of serum ALT during rhGH treatment over time by a generalized additive mixed model adjusted for age and gender (n = 253). (a) Description of serum ALT. (b) Change in ALT from baseline. ALT: alanine aminotransferase; rhGH: recombinant human growth hormone.

As shown in Figure 1, change of serum ALT during rhGH treatment over time was described by a generalized additive mixed model adjusted by age and gender.  Figure 1(a) depicted the distribution of serum ALT at different follow-up times. Meanwhile, mean values of the change in ALT from baseline displayed that, during the early stages of rhGH treatment, serum ALT level indicated a temporary upward trend, but it gradually decreased subsequently (*β*: -0.16; 95%CI: -0.23, -0.09;* P* < 0.001) (Figure 1(b)).

## 4. Discussion

This retrospective study investigated the association between the ALT and GH in short children and adolescents. The results showed that the GH peak level was independently and negatively related to serum ALT. Moreover, mean values of the change in ALT from baseline displayed that although serum ALT level indicated a temporary upward trend during the early stages of rhGH treatment, it gradually decreased over time.

There are various methods for screening and diagnosis of NAFLD. Among them, imaging examinations, including ultrasound, CT, and MR, play important roles. Recently, Esterson et al. [5] reviewed and discussed the sensitivity, specificity, advantages and limitations of the above examinations, which may not be suitable for large-scale epidemiological studies due to their characteristics, including required time and cost. Fortunately, serum ALT is cheaper and more accessible, and in numerous national guidelines, the use of ALT is recommended to screen for NAFLD in children [23, 24]. According to the literature for the definition of ALT elevation [6, 7, 25, 26], the proportion of ALT elevation was relatively high in this study. However, it should be stressed that the data from liver biopsy specimens in children with NAFLD showed that significant histological abnormalities can occur with normal or mildly elevated ALT levels [7]. Thus, the appropriate ALT cutoff values must be further explored and defined.

NAFLD is a multifactorial disease involved in various pathophysiological factors. Numerous studies have shown that dysfunction of the hypothalamic-pituitary axes might be an important etiology for NALFD [27]. GH is mainly synthesized and secreted in the anterior pituitary gland, which can regulate not only the body's growth but also its metabolism and composition [28, 29]. Many studies have shown that low levels of GH are causally related to hepatic lipid accumulation and promote the occurrence and progression of NAFLD. However, these studies mainly focused on the effects of GH in the adult liver [30]. Notably, some studies have demonstrated the roles of GH in pediatric patients. Gilliland et al. [15] reported a pediatric case of NASH secondary to panhypopituitarism from craniopharyngioma and found that GH replacement therapy led to rapid and complete resolution of hepatic steatosis. Similarly, Fujio et al. [14] showed that GH therapy could reduce transaminase levels in a pediatric patient with NAFLD associated with hypopituitarism. Therefore, the data suggest that NAFLD should be identified and screened in pediatric patients with GHD.

In our study, we found that serum ALT and incidence of ALT elevation significantly increased in short children and adolescents with the low GH state, and univariate analysis showed a negative correlation between ALT and GH levels. It is well known that obesity is a major risk factor for NAFLD [31]. BMI is useful indicator for evaluating obesity and nutritional status [32], and there is a strong relationship between BMI and prospectively recorded diagnoses of NAFLD [33]. We found that there was a close relationship between ALT and BMI. However, GH peak level was still independently related to ALT after adjusting for BMI. In addition, GH stimulates IGF-1 generation mainly in the liver [34], and as an important member of the GH/IGF-1 axis, there was a negative correlation between IGF-1 and NAFLD [35]. Low serum IGF-1 level was associated with increased histologic severity [36]. Inconsistent with previous studies, univariate analysis showed that IGF-1 was positively related to ALT, and GH fit well in our regression model after adjusting for IGF-1. The above data suggest that GH level might be more correlated with ALT than other variables. Moreover, some studies showed that the decrease in ALT might be a valid monitoring biomarker of histologic improvement of NAFLD [37, 38]. In this study, the results displayed that the ALT level was gradually decreased during rhGH therapy, which suggest that GH probably has potential values in prevention and treatment of NAFLD in short children and adolescents with the low GH state.

There are several limitations to our current study. Serum ALT was used as an independent variable to screen the suspected NAFLD, but imaging examinations were still needed to diagnose NAFLD. In fact, abdominal ultrasound was used to diagnose NAFLD in our study, but the results showed that NAFLD was not discovered. As ultrasound is not ideal for detecting the early stages of NAFLD [5], a more accurate method should be used to assess histologic severity in the liver in the future.

In conclusion, GH secretion level was strongly correlated with ALT in short children and adolescents, and rhGH therapy could reduce ALT level over time. This suggests that it will be important to screen for NAFLD and use rhGH treatment in short stature, especially when caused by GHD.

## Figures and Tables

**Table 1:** Basic characteristics of the subjects.

Variables	Total (n = 670)
Sex (Male, n, %)	472 (70.5)
Age (years)	10.2 ± 3.5
Height (cm)	125.6 ± 18.0
HT SDS	-2.67 ± 0.57
BMI (kg/m^2^)	16.85 ± 2.81
SBP (mmHg)	104.4 ± 11.8
DBP (mmHg)	62.7 ± 8.9
TC (mmol/L)	3.83 ± 0.72
TG (mmol/L)	0.72 ± 0.33
HDL-c (mmol/L)	1.62 ± 6.18
LDL-c (mmol/L)	2.09 ± 1.14
FPG (mmol/L)	4.76 ± 0.69
Cr (*μ*mol/L)	39.33 ± 14.74
UA (*μ*mol/L)	262.64 ± 80.23
GH peak (ng/mL)	8.80 ± 6.17
IGF-1(ng/ml)	186.97 ± 147.99
ALT (U/L)	16.09 ± 8.20
Increased ALT (n, %)	60 (9.0)

HT SDS: the standard deviation score of height; BMI: body mass index; SBP: systolic blood pressure; DBP: diastolic blood pressure; TC: total cholesterol; TG: triglycerides; HDL-c: high-density lipoprotein cholesterol; LDL-c: low-density lipoprotein cholesterol; FPG: fasting plasma glucose; UA: uric acid; GH: growth hormone; IGF-1: insulin-like growth factor-1; ALT: alanine aminotransferase.

**Table 2:** Comparison of anthropometric and biochemical characteristics.

Variables	GH peak			*P *value
	<5 (n = 182)	5-10 (n = 271)	≥10 (n = 217)	
Age (years)	10.3 ± 3.2	10.1 ± 3.4	10.3 ± 3.9	0.634
BMI (kg/m^2^)	17.97 ± 3.74	16.56 ± 2.15	16.28 ± 2.34	<0.001
SBP (mmHg)	105.0 ± 12.1	103.6 ± 11.5	104.9 ± 12.0	0.370
DBP (mmHg)	63.3 ± 8.4	62.24 ± 8.3	62.7 ± 10.0	0.489
TC (mmol/L)	3.93 ± 0.76	3.82 ± 0.72	3.76 ± 0.69	0.080
TG (mmol/L)	0.80 ± 0.43	0.70 ± 0.29	0.67 ± 0.25	<0.001
HDL-c (mmol/L)	1.43 ± 0.44	1.97 ± 9.71	1.35 ± 0.27	0.507
LDL-c (mmol/L)	2.28 ± 1.99	2.02 ± 0.59	2.02 ± 0.52	0.041
FPG (mmol/L)	4.71 ± 0.70	4.82 ± 0.76	4.73 ± 0.58	0.202
Cr (*μ*mol/L)	38.66 ± 7.69	40.27 ± 20.37	38.73 ± 10.10	0.403
UA (*μ*mol/L)	271.41 ± 71.64	247.70 ± 75.90	272.49 ± 89.61	0.003
IGF-1 (ng/ml)	182.56 ± 192.87	177.24 ± 108.56	202.73 ± 148.13	0.192
ALT (U/L)	17.85 ± 9.58	15.18 ± 7.83	15.76 ± 7.13	0.002
Increased ALT (n, %)	26 (14.29)	20 (7.38)	14 (6.45)	0.012

BMI: body mass index; SBP: systolic blood pressure; DBP: diastolic blood pressure; TC: total cholesterol; TG: triglycerides; HDL-c: high-density lipoprotein cholesterol; LDL-c: low-density lipoprotein cholesterol; FPG: fasting plasma glucose; UA: uric acid; GH: growth hormone; IGF-1: insulin-like growth factor-1; ALT: alanine aminotransferase.

**Table 3:** Correlation between the ALT and GH peak level by a univariate analysis.

Variables	*β* (95%CI)	*P* value
Age	0.36 (0.19, 0.54)	<0.001
BMI	0.59 (0.38, 0.81)	<0.001
SBP	0.12 (0.07, 0.17)	<0.001
DBP	0.12 (0.05, 0.19)	<0.001
TC	0.26 (-0.63, 1.15)	0.573
TG	2.03 (0.09, 3.97)	0.041
HDL-c	-0.04 (-0.14, 0.07)	0.466
LDL-c	0.07 (-0.49, 0.63)	0.804
FPG	0.36 (0.51, 1.23)	0.414
Cr	0.02 (-0.02, 0.06)	0.416
UA	0.01 (0.00, 0.02)	0.008
GH peak	-0.12 (-0.22, -0.02)	0.023
IGF-1	0.00 (0.00, 0.01)	0.024

BMI: body mass index; SBP: systolic blood pressure; DBP: diastolic blood pressure; TC: total cholesterol; TG: triglycerides; HDL-C: high-density lipoprotein cholesterol; LDL-C: low-density lipoprotein cholesterol; FPG: fasting plasma glucose; UA: uric acid; GH: growth hormone; IGF-1: insulin-like growth factor-1; ALT: alanine aminotransferase.

## Data Availability

The data used to support the findings of this study are available from the corresponding author upon request.
